# Validation of Miniaturized Particulate-Matter Real-Time Samplers for Characterizing Personal Polycyclic Aromatic Hydrocarbon Exposure

**DOI:** 10.4172/2155-9872.1000403

**Published:** 2018-04-11

**Authors:** Beizhan Yan, Masha Pitiranggon, James Ross, Thomas Arthen-Engeland, Andreas Stelter, Steven N. Chillrud

**Affiliations:** 1 Lamont-Doherty Earth Observatory of Columbia University, Palisades, NY, USA; 2 Bruker Daltonik GmbH, Bremen, Germany

**Keywords:** Ionization, Pollutants, Chromatograph, Organic compounds, Mass spectrometry

## Abstract

This study validates the analysis of polycyclic aromatic hydrocarbons (PAHs) in microgram levels of particulate matter (PM) collected on filters by two low-flow rate, real-time monitors, microPEM™ and microAeth^®^. Particle-associated PAHs were analyzed by a coupling of a gas chromatograph to a sensitive, atmospheric-pressure laser ionization-mass spectrometer. Air particulate samples were collected over the course of one or two days in the living room of a fourth-floor apartment in New York City. Three types of samplers, the two aforementioned personal samplers and a high-flow rate pump (4 liters per minute), were operated side by side, and three samples of each type were collected during each sampling period. Intrasampler agreement as measured by relative standard deviation (RSD) was within 1% to 18%. After background subtraction, total PAH measured by all three sampler types had good agreement (R=0.99). This ability to accurately characterize personal PAH exposure in archived filters collected by these real-time samplers could provide additional important PAH exposure information that can benefit many environmental health studies using these monitors.

## Introduction

Personal monitors are widely accepted as the gold standard in air pollution exposure assessment. However, traditional personal exposure monitors tend to be too cumbersome, noisy and labor-intensive, and do not provide near-real-time measurements of key analytes, limiting most personal exposure studies to motivated cohorts of adolescents and adults [1,2]. Recently some miniaturized realtime personal monitors (e.g., microPEM™ by RTI International, Airbeam by HabitatMap, SidePak™ by TSI Incorporated) of particulate matter (PM) based on optical methods have been developed and used in epidemiology and environmental science studies [3–7]. The minimization of size, power, and noise, permits the use of these personal monitors on most individuals, including young children, the elderly and occupational workers, without disruption of their normal activities [8–10]. Among these personal monitors, several have a filter placed before the pump and the purpose of these filters varies. For example, the Teflon filter used in microPEM™ not only protects the pump from aerosol particles, but also can be gravimetrically weighed to calibrate the realtime data [5]. For microAeth^®^, a real time black carbon (BC) monitor, the optical attenuation caused by deposited particles on the filter is measured to determine BC loading [11]. If the filters in those samplers can be changed before each deployment, these spatial- and temporal- integrated samples have the potential to characterize PM exposure.

It will be highly cost-effective and scientifically important if key pollutant levels can be reliably quantified in these archived filters. However, minimization also inevitably results in a low flow pump that collects low mass particle samples. The AE51 model of a microAeth^®^ unit is about 250 grams, 117 mm L × 66 mm W × 38 mm D in size and can be run at a flow rate of less than 0.2 liter per minute (LPM) with pumped air passing through a 3 mm spot on a filter strip [11,12]. The filter strip is made of T60 Teflon-coated borosilicate glass fiber filter material. The microPEM™ is about 240 grams, runs at a flow rate of 0.4-0.5 LPM with pumped air passing through a 3 micron porosity Teflo membrane filter (Pall Corp, Port Washington, NY). To extend battery life, both microAeth^®^ and microPEM™ can be operated at a duty cycle <100% (i.e., pump and/or sensor can be cycled on and off at different time intervals). Depending on airborne PM level, the flow rate and duty cycles used during the sampling, the mass collected on filters can range from less than 1 μg to tens of μg. In contrast, traditional personal monitors typically collect 50 μg to a few hundred μg of PM2.5 since much higher flow rates (e.g., 4 LPM) are used [13]. In our lab, we have established analytical approaches to measure additional pollutants in filters collected by traditional samples, e.g., black carbon by an optical method (limit of detection (LOD) is about 0.5 μg) [14] and metals using X-ray fluorescence spectrometry [15] (LOD has not been determined in our lab).

One important class of pollutants is polycyclic aromatic hydrocarbons (PAHs) since numerous studies have suggested that exposures to PAHs are negatively associated with a variety of health outcomes, including asthma symptoms [16]. Lines of evidence also suggest that certain PAH compounds are suspected or known carcinogens and mutagens [17,18]. PAH levels in New York City air samples are typically on the order of micrograms per gram of PM [19], therefore only picogram (10^−12^ gram) levels of PAHs can be collected by these miniaturized samplers. This pushes the analytical limits of most traditional mass spectrometry methods, which normally requires around 10 pg to be above detection limits for an individual PAH compound [20]. For a robust quantification, at least 30 pg of the compound would be needed, going by the convention that the limit of quantitation is three times the detection limit [21,22].

A novel analytical instrument, the gas-chromatograph–atmospheric-pressure laser ionization-time of flight mass spectrometer (GC-APLI-TOFMS), was recently developed by Schiewek et al. [23], and to our knowledge it is the most sensitive instrument for aromatic compound analysis [24]. APLI is based on resonantly enhanced multi photon ionization at atmospheric pressure and selectively ionizes analytes with longer lifetimes in electronically excited states for the absorption of additional photons. The step-wise 2-photon absorption of APLI reduces the ionization of solvent and matrix molecules and therefore increases analytical selectivity and sensitivity [24–26]. This increased sensitivity would potentially allow for the measurement of PAHs collected by personal air samplers on the time range of a few hours at levels found in typical US cities and homes.

Our goal in this study is to validate the usage of archived filters collected by these miniatured samplers for PAH analysis. We use GC-APLI-TOFMS to examine the specificity, sensitivity, and range necessary for accurate quantification of individual PAH compounds in the amounts collected on these mini-filter matrices. In this study, results from two different personal monitors (microPEM™ and microAeth^®^) will be compared to a conventional sampler.

## Materials and Methods

### Calibration

Our PAH calibration standards consisted of a mix of 16 parental PAHs (Restek (Bellefonte, PA) SV Calibration Mix, Cat. #31011), five alkyl-PAHs (2,6-dimethylnaphthalene, 2-methylphenanthrene, 3,6-dimethylphenanthrene, 1-methylpyrene, and 6-methylbenz[a]anthracene) from Accustandard (New Haven, CT), and two external standard compounds (acenaphthene-D10 and perylene-D12) from Accustandard (New Haven, CT). Six different concentrations of this mix were used for calibration, and each were spiked with equal amounts (1.0 × 10^5^ femtograms/μl (fg/μl)), of five different internal standards: acenaphthylene-D8 (Cambridge Isotope Laboratories, Tewksbury, MA), anthracene-D10 (Supelco, St. Louis, MO), terphenyl-D14 (Supelco, St. Louis, MO), benzo[a]pyrene-D12 (SPEX CertiPrep, Metuchen, NJ), and indeno[c,d-1,2,3]pyrene-D12 (Cambridge Isotope Laboratories, Tewksbury, MA). The six different concentrations were 6.25, 12.5, 25, 50, 80, and 100 fg/μl. Calibration standards at each concentration were run in triplicate on GC-APLI. Calibration curves were made using averages of the triplicate runs.

### Sample collection

Air particulate samples were collected over the course of eleven 24-hour sampling periods and one 48-hour sampling period in the living room of a fourth-floor apartment in the Upper West Side of Manhattan in New York City, NY. Three types of samplers were operated side by side and three samples of each type were collected during each sampling period. Two types of samplers collected PM2.5 on Teflon filters, one from a black box pump (BB) with conventional cyclone (model GK 2.05, BGI, Inc.) at 4 ± 0.4 L/min and the other from a microPEM™ (μPEM) personal exposure monitor (RTI International, Research Triangle Park, NC) operated at 0.4 ± 0.04 L/min. The third sampler was a microAeth^®^ (μAeth) personal black carbon (BC) monitor (AethLabs, San Francisco, CA), which collected dust samples on Teflon-coated borosilicate glass fiber filters (T60) at 0.1 ± 0.01 L/min. With the exception of one μPEM that was excluded from the analysis here, the relative standard deviation of the flow rates measured at the start and end of each sampling period stayed within 0% to 7%. One field blank for each type of sampler was obtained by setting up the sampler as usual, but without turning on their respective pumps. All filters were pre-cleaned by dichloromethane in an ultrasonic bath prior to sampling.

### PAH extraction

Organic extracts from each filter were obtained via ultrasonic extraction in 1 mL of 9:1 dichloromethane:methanol (v/v) for 60 minutes. Each sample was spiked with 10 ng of acenaphthene-d10 and perylene-d12 before extraction as external standards. Following extraction, each sample underwent silica column cleanup to remove polar organic compounds [27]. Each silica column consisted of 0.720 g of silica gel topped off with a thin layer of sodium sulfate. Dichloromethane was used as the eluent, and approximately 4 mL of eluent was collected from each column. The samples were then concentrated under N2 gas and spiked with an internal standard mix containing: acenaphthylene-d8, anthracene-d10, terphenyl-d14, benzo[a]pyrene-d12, and indenopyrene-d12. The conventional cyclone samples were spiked with 20 ng of the internal standard mix. All other samples were spiked with 10 ng of internal standard. Extracts were stored at 4°C until further analysis.

### Analysis by GC-APLI

The organic filter extracts were analyzed using a Bruker 450-GC and a Compact time-of- flight mass spectrometer (Bruker Daltonics, Bremen, Germany). 2 μl of each sample were injected in splitless mode with the inlet temperature at 275°C. The GC column was a Restek Rxi-PAH column (40 m length, 0.18 mm ID, 0.07 μm df). The column oven was initially held at 40°C for 1 minute, then increased to 110°C at 40°C/min, held for 1 minute, increased again to 210°C at 37°C/min, where the ramp rate slowed to 3°C/min up to 260°C, finally ramping at a rate of 11°C/min up to 350°C, where it was held for 8.7 minutes. Helium was used as the carrier gas at a constant flow rate of 1.4 mL/min. The mass spectrometer was operated with a GC-APLI II source in atmospheric pressure laser ionization mode, using a diode-pumped solid state (DPSS) laser (CryLas GmbH, Berlin, Germany) operated at 266 nm with a 200 Hz repetition rate and 60 μJ per pulse [28]. Masses 50-500 were scanned at an acquisition speed of 2 Hz. The capillary was set at −1000 V with an end plate offset of 0 V. Nebulizer gas was supplied at 3 bar, and the drying gas had a flow rate of 2.5 L/min at 250°C.

## Results and Discussion

### GC-APLI analysis and quantitation

**Figure 1:**
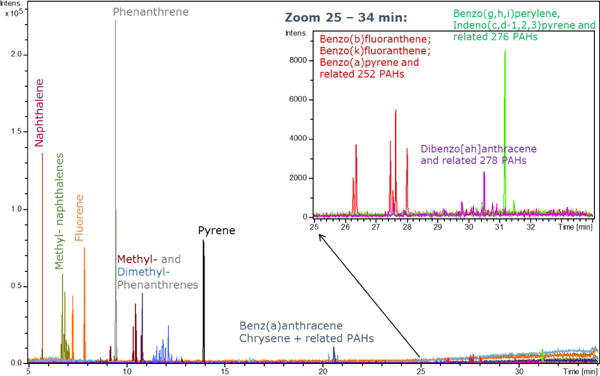
Example extracted ion chromatogram (EIC) traces of target PAHs in GC-APLI chromatogram of a uAeth filter extract.

GC-APLI proved to be a highly sensitive instrument for ultra-trace level measurement of PAHs in low mass PM samples collected from personal samplers (Figure 1), consistent with other reports [24,28]. Figure 1 shows a chromatogram of a typical PAH profile from a sample with anthropogenic contributions, as indicated by the typical pattern of methyl- and dimethyl- substituted phenanthrenes. Additionally, several isomers of benzofluoranthenes and dibenzanthracenes were properly separated in the chromatograms, indicating the variety of PAHs observed in these samples.

The analytical working range investigated was 6 to 100 fg/μl for 20 PAHs, although real the analytical working range is expected to be larger. Of those compounds that were above the detection limit, individual PAH concentrations were estimated to range from 0.086 fg/μl to 1800 fg/μl for μAeth samples and 3.1 fg/μl to approximately 1500 fg/μl for μPEM samples. his means that GC-APLI was able to detect individual PAHs down to 0.17 fg total on-column injection. Most conventional mass spectrometry methods need an on-column injection of 10 pg (1 × 10^4^ fg) to overcome detection limits for individual PAHs [20]. Since the samples involved in this study pertain to particle-associated compounds, PAHs with m/z greater than 202 were the most relevant, and analysis was limited to these high molecular weight PAHs: 1-methylpyrene, benz[a]anthracene, chrysene, 6-methylbenz[a]anthracene, benzo[b]luoranthene, benzo[k]luoranthene, benzo[a]pyrene, dibenz[ah]anthracene, benzo[ghi]perylene. he sum of these PAHs is referred to as TPAH in the remainder of this text.

**Figure 2:**
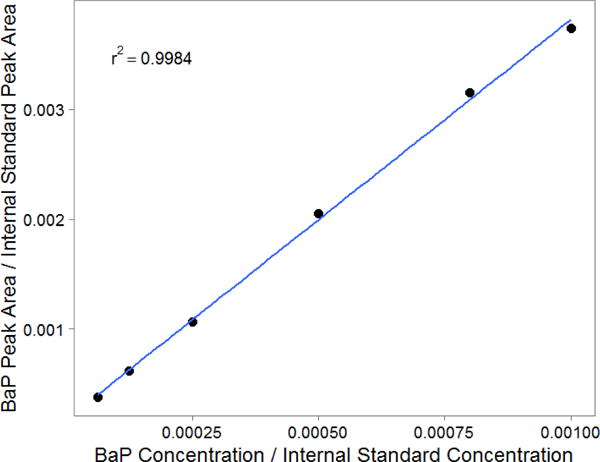
Benzo[a]pyrene (BaP) calibration curve. All PAHs in the sampler validation analysis had an r-squared of 0.99 and above.

The calibration curves for all PAHs used in sampler agreement analysis had r-squared values of 0.99 and above (Figure 2, Table S1). Indeno[c,d-1,2,3]pyrene was excluded due to issues with its calibration; it was eluting at the same retention time as its internal standard, indeno[c,d-1,2,3]pyrene-D12, which was present at a much higher concentration than indeno[c,d-1,2,3]pyrene in all calibration standards and samples. Signal disturbance from the internal standard may have resulted in unreliable peak quantification. See Supplemental Information for low molecular weight PAH and indeno[c,d-1,2,3]pyrene calibration results.

### Sampler comparison

Due to time constraints and sample availability, 2-3 replicates of each sampler type from 4 sampling periods were selected to be analyzed on APLI. Background correction was performed on TPAH measurements from all samples by subtracting the TPAH measured on each samplers’ respective field blank. This background-corrected data is presented in Table 1. Due to the extremely low mass of PAHs (fg) collected onto the filters of miniaturized samplers, there are special requirements for obtaining reliable data: 1) a clean lab equipped with laminar flow hood fitted with HEPA filter to be used for filter handling, 2) a clean organic lab to handle solvents and PAH analysis, 3) subtracting the PAHs measured in field blank samples. Relatively high PAH background was observed in the T60 filter, and without background correction, PAH levels will be overestimated.

**Figure 3:**
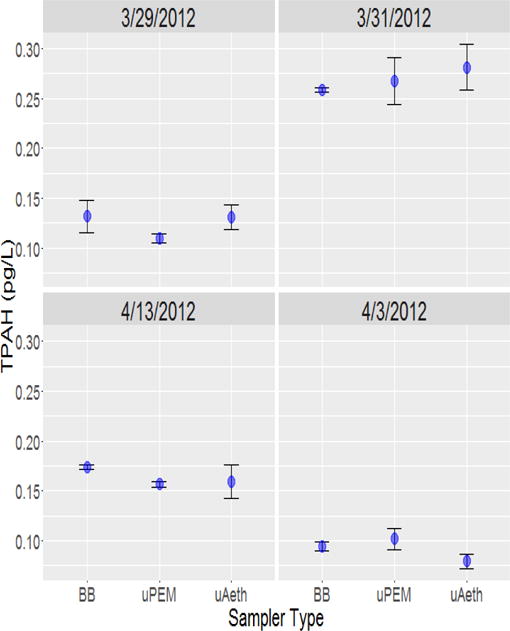
Comparison of TPAH concentrations by 3 different units, black box pump (BB), microPEM™ (uPEM), and microAeth^®^ (uAeth). The points represent the mean TPAH measurement for each sampler on each sampling day with error bars representing the standard error of the mean. N=2 for every sampler on each sampling day except for the uAeth on 3/29/2012, 4/3/2012, and 4/13/2012, where N=3.

After background subtraction, side-by-side deployment of μAeth, μPEM, and BB samplers revealed acceptable reproducibility of sampler replicates on each sampling day (Figure 3). Intrasampler agreement as measured by relative standard deviation (RSD) was within 1% to 18%. For BB pumps, the mean RSD for TPAH in each sampling period was 7% ± 4%, with a mean absolute standard deviation of 9 ± 5 fg/L. For μAeth personal samplers, the mean RSD for TPAH in each sampling period was 16% ± 1%, with a mean absolute standard deviation of 24 ± 4 fg/L. For μPEM personal samplers, the mean RSD for TPAH in each sampling period was 9% ± 3%, with a mean absolute standard deviation of 15 ± 7 pg/L.

**Figure 4:**
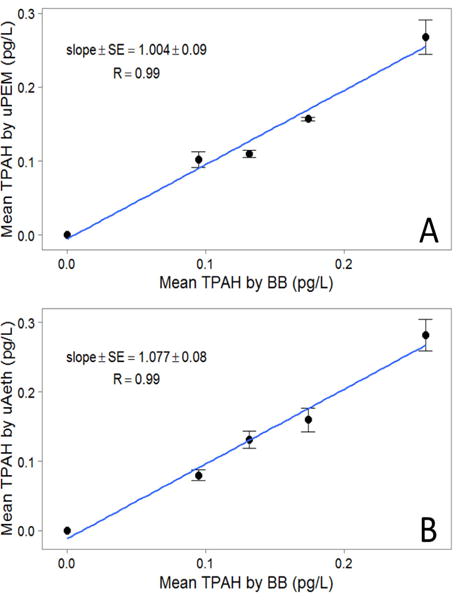
**(A)** The linear association of PAH measurements by microPEM™ personal samplers (uPEM) and conventional black box pumps (BB). Error bars represent the standard error of the mean TPAH measurements made by microPEM™. **(B)** The linear association of PAH measurements by microAeth^®^ personal samplers (uAeth) and conventional black box pumps (BB). Error bars represent the standard error of the mean TPAH measurements made by microAeth^®^. Points at the origin represent field blanks.

In addition to acceptable intrasampler agreement, intersampler agreement was also very good. μPEM samples had PAH concentrations that were consistent with samples collected by conventional BB pumps (Figures 3 and 4), and TPAH for each sampling period (N=4) were highly correlated (R=0.99; slope ± SE=1.004 ± 0.09). The mean RSD of TPAH among μPEMs and conventional cyclones in each sampling period was 7% ± 2%, with a mean absolute standard deviation of 10 ± 2 fg/L. There is also a very high correlation of microAeth^®^ TPAH and conventional pump TPAH (Figures 3 and 4) for each sampling period (R=0.99; slope ± SE=1.077 ± 0.08), and the mean RSD among microAeths^®^ and conventional cyclones was 6% ± 2%, with a mean absolute standard deviation of 9 ± 3 fg/L. Excluding dibenz[a,h]anthracene and those compounds that were below detection limit, intersampler agreement was also decent when looking at individual PAH concentrations as measured by the different samplers (Table 2). Dibenz[ah]anthracene had some of the lowest mass loadings of all the high molecular weight PAHs in these samples; it is possible that these mass loadings were not high enough for reliable quantitation. Otherwise, mean RSDs among microAeths^®^ and conventional cyclones ranged from 9% ± 3% for benzo[ghi]perylene to 29% ± 6% for benzo[a]pyrene. Similarly, among microPEMs™ and conventional cyclones, mean RSDs ranged from 9% ± 3% for benzo[ghi]perylene to 32% ± 13% for 1-methylpyrene.

In summary, this study validates the analysis of PAHs in archived PM samples collected by miniaturized personal exposure monitors if they have a filter to collect PM. Due to the low mass collected, a sensitive PAH analysis is required and our research shows that GC-APLI-TOFMS is a novel method for identifying and quantifying ultra-trace-levels of PAHs. To our knowledge, there is no other instrument available with the ability to accurately measure PAHs at such low levels. The ability to accurately quantify personal PAH exposure would greatly strengthen any epidemiological study aiming to determine the health effects of airborne pollutant exposure. Future studies should consider minimum sampling times necessary to collect adequate sample mass for reliable quantitation by running samplers at variable sampling times.

## Supplementary Material

Supplement

## Figures and Tables

**Table 1:** TPAH measurements made by conventional black box pump (BB), microPEM™ (uPEM), and microAeth^®^ (uAeth) in four different sampling periods

Date	Replicate #	TPAH by BB (Pg/L)	TPAH by uPEM (Pg/L)	TPAH by uAeth (Pg/L)
3/29/2012	1	1.15E-01	1.05E-01	1.56E-01
3/29/2012	2	1.48E-01	1.15E-01	1.21E-01
3/29/2012	3	na	na	1.16E-01
3/31/2012	1	2.57E-01	2.91E-01	3.04E-01
3/31/2012	2	2.61E-01	2.44E-01	2.59E-01
3/31/2012	3	na	na	na
4/3/2012	1	9.04E-02	9.15E-02	7.42E-02
4/3/2012	2	9.91E-02	1.13E-01	9.47E-02
4/3/2012	3	na	na	7.00E-02
4/13/2012	1	1.77E-01	1.59E-01	1.65E-01
4/13/2012	2	1.72E-01	1.54E-01	1.28E-01
4/13/2012	3	na	na	1.85E-01

**Table 2:** Mean percent relative standard deviations (standard error of the mean) for each individual PAH included in the sampler validation analysis.

	Black Box vs. microAeth Mean %RSD	Black Box vs. microPEM Mean %RSD
1-Methylpyrene	na	32 (13)
Benz[a]anthracene	na	24 (7)
Chrysene	na	16 (5)
6-Methylbenz[a]anthracene	na	na
Benzo[b]fluoranthene	25 (14)	25 (5)
Benzo[k]fluoranthene	22 (7)	13 (5)
Benzo[a]pyrene	29 (6)	11 (5)
Dibenz[ah]anthracene	109 (8)	56 (9)
Benzo[ghi]perylene	9 (3)	9 (3)
