# Supplementary material for: Validation of Miniaturized Particulate-Matter Real-Time Samplers for Characterizing Personal Polycyclic Aromatic Hydrocarbon Exposure
Source: J Anal Bioanal Tech. Author manuscript; Available in PMC 2018 Jul 10. (PMC6039126; doi:10.4172/2155-9872.1000403)
Supplement: Supplement [file NIHMS971067-supplement-Supplement.pdf]

Supplemental Information

Most PAHs with m/z of 202 and below, exhibited high blind values with the exception of 3,6-dimethylphenanthrene and 2-methylphenanthrene. One possible explanation is that our ultra-trace-level analysis is much more susceptible to interference from contamination in the laboratory air. PAHs with high blind values are defined, here, as those having calibration curves with y-intercepts that are greater than 30% of the highest y-value in the calibration curve. The following are PAHs which exhibited high blind values: naphthalene, 2,6-dimethylnapthalene, acenapthene, fluorene, phenanthrene, anthracene, fluoranthene, pyrene, and indeno[1,2,3-c,d]pyrene (Table S1). All PAHs for which blind values exceeded the threshold of 30% were not included in analyses of sampler agreement in this study. Because of the focus on particle-associated compounds, only the high molecular weight PAHs (m/z>202) were relevant to this study, and, with the exception of indeno[c,d-1,2,3]pyrene, PAHs with m/z greater than 202 did not exhibit high blind values. As mentioned in the main text, the poor calibration curve for indeno[c,d-1,2,3]pyrene may be due to interference from its co-eluting internal standard, which was added at a much higher concentration. The high blind values observed for many of the LMW PAHs may be due to contamination in the laboratory air. Cleaner lab environments may be necessary for this type of ultra-trace level analysis.

	**Y-intercept**	**Maximum y-value**	**R-squared**
**Naphthalene**	0.0073	0.0182	0.8267
**2,6-Dimethylnapthalene**	0.0061	0.016	0.9545
**Acenaphthene**	0.0062	0.0122	0.9948
**Fluorene**	0.0237	0.0419	0.9715
**Phenanthrene**	0.0344	0.0811	0.8079
**2-Methylphenanthrene**	0.0006	0.0334	0.9456
**3,6-Dimethylphenanthrene**	0.0002	0.0302	0.9979
**Anthracene**	0.0008	0.0022	0.9685
**Fluoranthene**	0.0004	0.0008	0.847
**Pyrene**	0.0139	0.0242	0.8562
**1-Methylpyrene**	0	0.0064	0.999
**Benz[a]anthracene**	0.0021	0.0071	0.9994
**6-Methylbenz[a]anthracene**	0.0002	0.0107	0.9961
**Chrysene**	0.0003	0.0045	0.9902
**Benzo[b]fluoranthene**	0.0001	0.0008	0.9934
**Benzo[k]fluoranthene**	0.0001	0.0029	0.9985
**Benzo[a]pyrene**	0.0002	0.0037	0.9984
**Indeno[1,2,3-c,d]pyrene**	0.0016	0.0016	0.5656
**Dibenz[a,h]anthracene**	0.0004	0.0077	0.9999
**Benzo[g,h,i]perylene**	0.0004	0.007	0.9986
